# Atorvastatin Plus Low-Dose Dexamethasone May Be Effective for Leukemia-Related Chronic Subdural Hematoma but Not for Leukemia Encephalopathy: A Report of Three Cases

**DOI:** 10.3389/fonc.2021.628927

**Published:** 2021-07-15

**Authors:** Jiangyuan Yuan, Ying Li, Xuanhui Liu, Meng Nie, Weiwei Jiang, Yibing Fan, Tangtang Xiang, Hanhua Wang, Wei Quan, Chuang Gao, Jinghao Huang, Shuo An, Yongxin Ru, Qiufan Zhou, Jianning Zhang, Rongcai Jiang

**Affiliations:** ^1^ Department of Neurosurgery, Tianjin Medical University General Hospital, Tianjin, China; ^2^ Tianjin Neurological Institute, Key Laboratory of Post-Neuroinjury Neuro-Repair and Regeneration in Central Nervous System, Tianjin Medical University General Hospital, Ministry of Education, Tianjin, China; ^3^ Department of Clinical Laboratory, Hospital of Hematology, Chinese Academy of Medical Sciences, Tianjin, China; ^4^ Department of Hematology, Tianjin Medical University General Hospital, Tianjin, China

**Keywords:** chronic subdural hematoma, leukemia, leukemia encephalopathy, atorvastatin, dexamethasone

## Abstract

We are not aware of any reports regarding conservative treatment for leukemia-related chronic subdural hematoma (CSDH). We report our experience with 3 men who were admitted with subdural masses and abnormal leukocyte counts. In two patients, leukemia and CSDH were confirmed on the basis of medical records, mild head trauma, and neuroimaging features. Both patients experienced reduced CSDH and neurological symptoms after receiving atorvastatin (20 mg/day) plus low-dose dexamethasone. However, this combined conservative treatment was ineffective in the third patient, who was diagnosed as having leukemia and showed an increased hematoma volume after two weeks of therapy. Magnetic resonance imaging findings suggested dural metastasis, which prompted a switch from statin-based conservative treatment to chemotherapy. Complete remission of the leukemia and resolution of the subdural mass were observed after chemotherapy, which supported a diagnosis of leukemia encephalopathy. The 5-month follow-ups did not reveal CSDH relapse in all 3 cases. Thus, atorvastatin-based conservative treatment may be effective for leukemia-related CSDH but not for leukemia encephalopathy.

## Introduction

Chronic subdural hematoma (CSDH) is a common neurological disease that generally develops after a head trauma (1–3). The formation of CSDH is thought to be related to the injury of the bridging vessels that connect the brain to the dural venous sinuses. This allows blood to collect in the subdural space, which induces chronic inflammation, the continuous release of angiogenesis-related cytokines, and the formation of highly permeable capillaries. Blood constantly seeps into the subdural space from these immature vessels, which causes the chronic hematoma (4–6). Cases of CSDH generally involve elderly patients and can co-occur with various neoplasms, including leukemia (7–9). However, there are few reports regarding leukemia-related CSDH, its potential etiology, and conservative treatment.

In this context, if leukemia is effectively treated and the CSDH persists, the underlying mechanisms and treatment strategies would be similar to those in cases of trauma-related CSDH. However, it is also possible that the subdural mass might be directly caused by leukemia encephalopathy, which would suggest that its pathological characteristics and treatment strategies would differ from those of trauma-related CSDH. Burr-hole drainage is the primary treatment for CSDH, although it is associated with unsatisfactory rates of relapse and mortality (10, 11). Furthermore, the current chemotherapeutic protocols for leukemia are still associated with a poor prognosis and abnormal blood parameters, which may motivate patients to select non-surgical conservative therapy if they have CSDH and leukemia (12). Unfortunately, there is no clearly effective conservative treatment for CSDH in patients with leukemia. We confirmed that statin-based conservative treatment is effective for conventional CSDH on the basis of findings from our previous clinical trials (13, 14). In this report, we describe the effectiveness of a statin-based conservative treatment for CSDH in patients with leukemia.

Clinical studies have indicated that atorvastatin, which is a 3-hydroxy-3-methylglutaryl-coenzyme A reductase inhibitor, can effectively treat CSDH by promoting vessel maturation and inhibiting intracapsular inflammation (15–17). Furthermore, preliminary findings from a randomized proof-of-concept clinical trial have indicated that atorvastatin plus low-dose dexamethasone treatment was effective in reducing hematoma and neurological symptoms in patients with CSDH (14). Thus, to save time and achieve better outcomes, it would be reasonable to use atorvastatin plus low-dose dexamethasone to treat leukemia-related CSDH, as this strategy was more effective than atorvastatin monotherapy. We report our experience with 2 patients who had leukemia and trauma-related CSDH and a third patient with leukemia encephalopathy that was diagnosed during hospitalization, who experienced different results from a combination of atorvastatin plus low-dose dexamethasone.

## Case Presentation

Informed consent for the publication of this report was obtained from the patients. All patients received a standard dose of atorvastatin (20 mg/day orally) plus low-dose dexamethasone (2.25 mg/day for the first 2 weeks, 1.5 mg/day during the third week, and then 0.75 mg/day during the fourth week).

### Case 1

**Figure 1:**
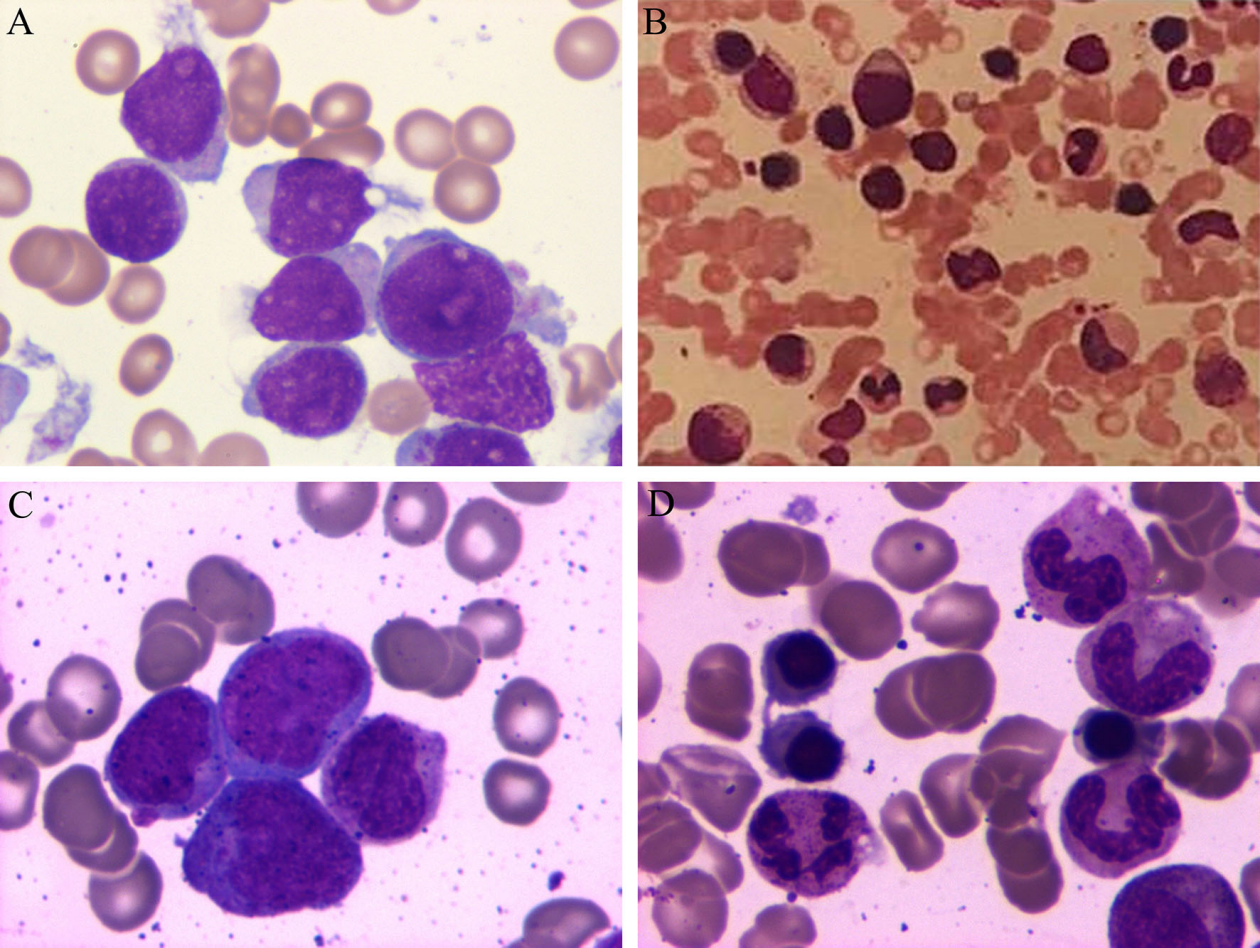
Periodic acid-Schiff staining of bone marrow aspirate from the 3 patients at the time of admission. **(A)** Patient 1 had acute myeloid leukemia, **(B)** Patient 2 had chronic myeloid leukemia, and **(C)** Patient 3 had acute myeloid leukemia. **(D)** Patient 3 experienced complete remission after the chemotherapy.

**Figure 2:**
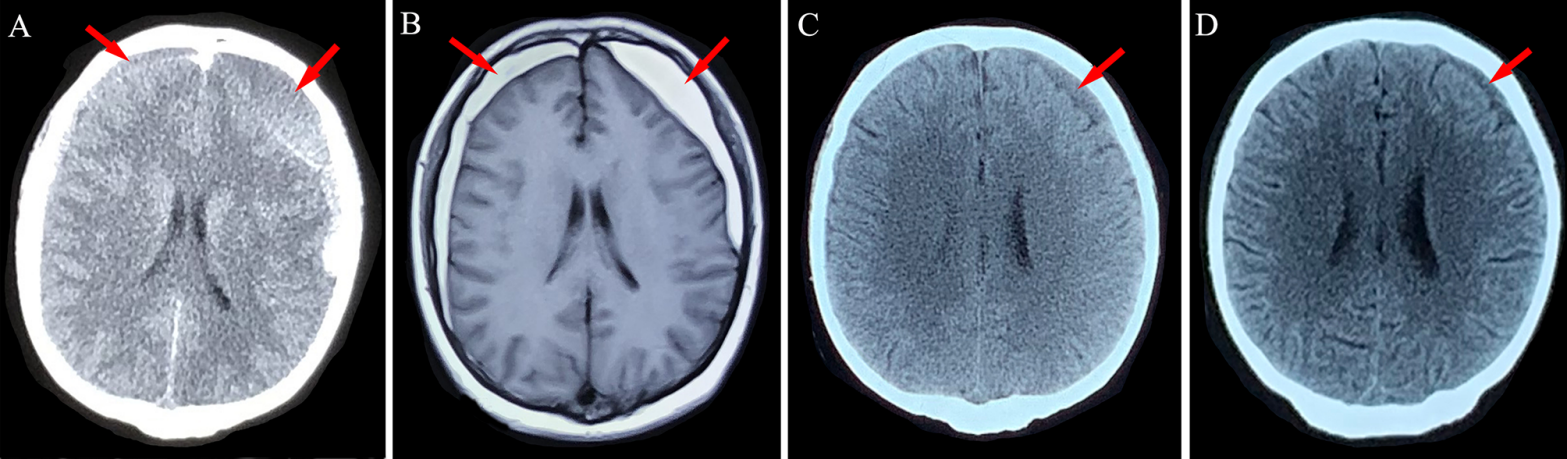
Neuroimaging findings for Patient 1. **(A)** Chronic subdural hematoma (red arrows) was detected in the bilateral frontotemporal lobes after head trauma. **(B)** After 4 weeks of atorvastatin treatment, the left-side hematoma was slightly absorbed. **(C, D)** Absorption of the hematoma after 8 weeks and 12 weeks of conservative treatment using atorvastatin plus low-dose dexamethasone.

A 28-year-old man with acute myeloid leukemia (AML) experienced head trauma during a minor collision while receiving chemotherapy with cytarabine plus decitabine (**Figure 1A**). One month later, the patient developed headache and confusion, and bilateral CSDH was detected during head computed tomography (CT) (**Figure 2A**). Treatment was immediately started using atorvastatin (20 mg/day), the patient’s symptoms were significantly relieved after 4 weeks, and magnetic resonance imaging (MRI) revealed slight absorption of the left-side hematoma (**Figure 2B**). Low-dose dexamethasone was added to the conservative treatment at 4 weeks, and CT at the 8-week follow-up revealed substantial absorption of the CSDH (**Figure 2C**). The dexamethasone treatment was subsequently stopped, but atorvastatin treatment was continued until week 12, at which point CT revealed that the hematoma had nearly disappeared (**Figure 2D**). The patient did not experience any adverse drug reactions during the conservative treatment, and no hematoma recurrence has been observed during the 6-month follow-up.

### Case 2

**Figure 3:**
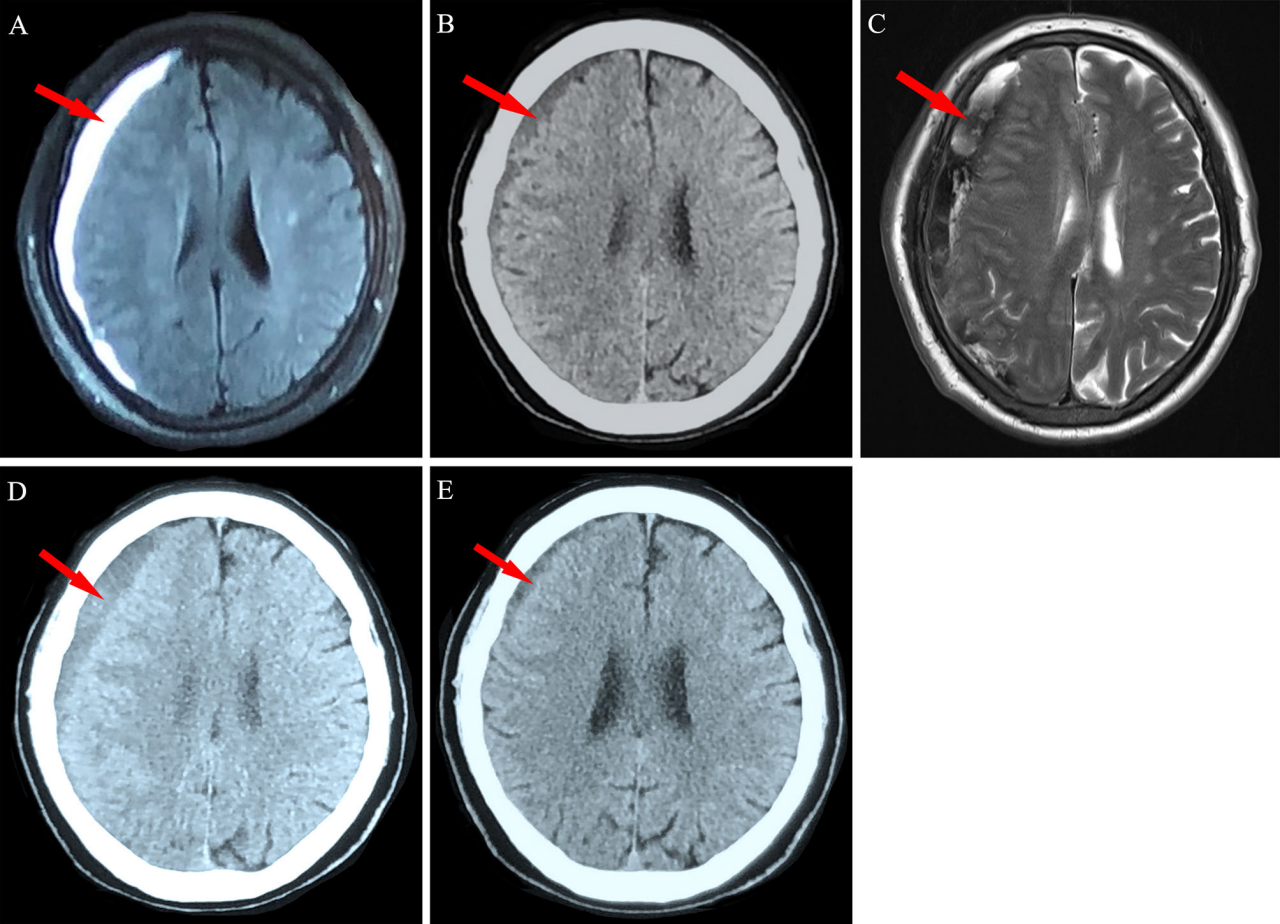
Neuroimaging findings for Patient 2. **(A)** A 72-year-old man with chronic myeloid leukemia was diagnosed as having right frontotemporal chronic subdural hematoma (red arrow). **(B)** The hematoma was significantly absorbed after 2 weeks of conservative treatment. **(C)** New bleeding in the subdural space was detected *via* magnetic resonance imaging at the 4-week follow-up. **(D)** Obvious absorption was observed after 10 weeks and **(E)** near disappearance was observed after 20 weeks of conservative treatment.

A 72-year-old man had a 2-year history of chronic myeloid leukemia (CML) but had not been receiving chemotherapy for approximately 1 year (**Figure 1B**). The patient developed CSDH after experiencing head trauma, which resulted in dizziness, headache, and left-limb weakness (**Figure 3A**). After 2 weeks of conservative treatment using atorvastatin plus dexamethasone, the hematoma was substantially absorbed, and the patient’s symptoms were significantly relieved (**Figure 3B**). However, the patient’s symptoms worsened during the 4^th^ week of treatment, at which point head MRI revealed new bleeding in the subdural space (**Figure 3C**). Conservative treatment was continued, although dexamethasone was stopped at the 6^th^ week and atorvastatin was stopped after approximately 20 weeks. At 10 weeks, CT revealed that the hematoma had been significantly absorbed (**Figure 3D**), and the hematoma gradually disappeared during the 5-month follow-up, with complete relief of the patient’s symptoms (**Figure 3E**). This patient also did not experience any adverse drug reactions or hematoma recurrence.

### Case 3

**Figure 4:**
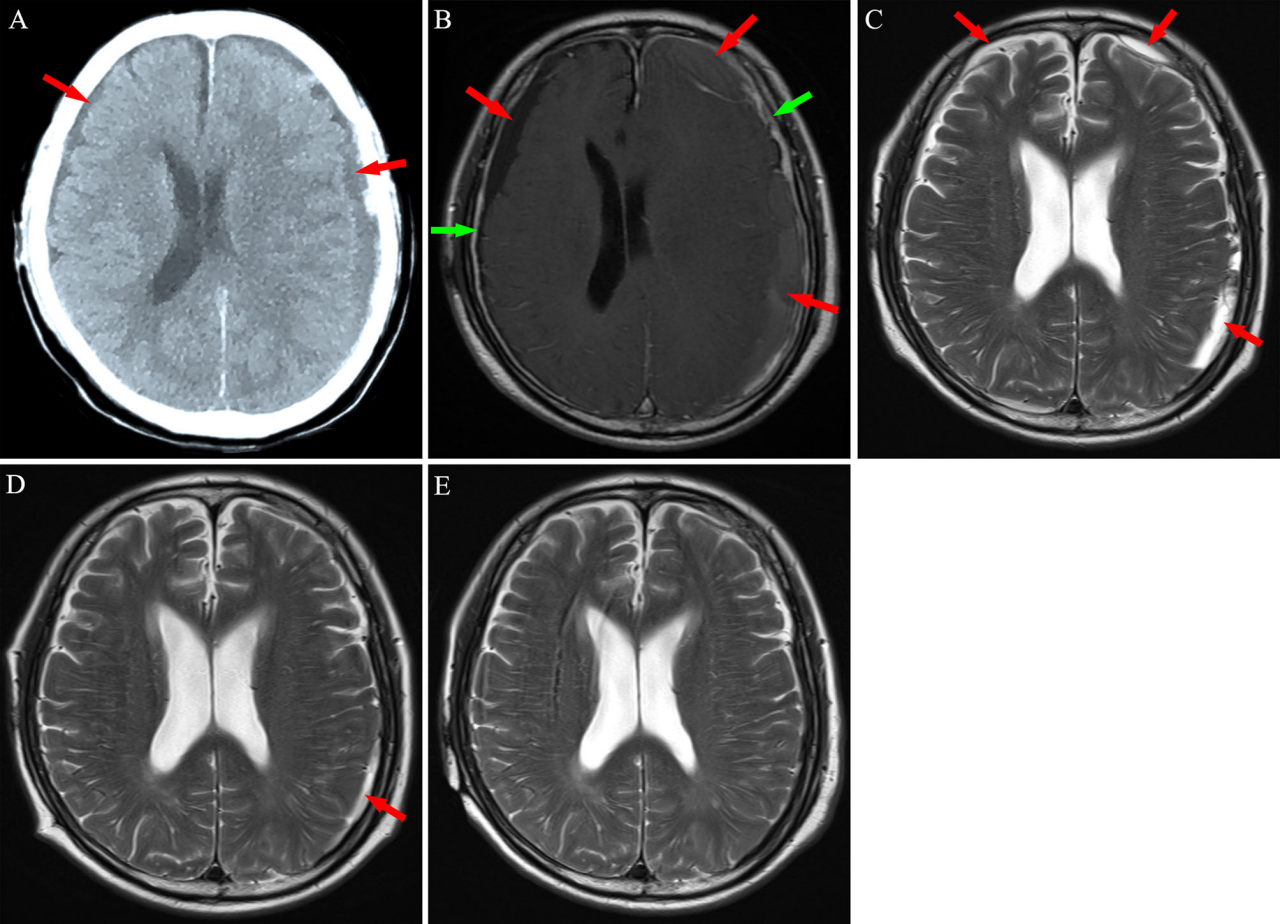
Neuroimaging findings for Patient 3. **(A)** Computed tomography revealed bilateral chronic subdural hematoma (red arrows). **(B)** After 2 weeks of atorvastatin treatment plus low-dose dexamethasone, magnetic resonance imaging revealed an increase in the hematoma, with significant enhancement of the dura mater (green arrows) post-injection of contrast agent. **(C, D)** After chemotherapy, subdural mass was significantly absorbed in 8-14 weeks. **(E)** A 5 months follow-up MRI exhibited no hematoma in the subdural space.

A 68-year-old man with a 1-month history of headache and dizziness was diagnosed as having CSDH (**Figure 4A**). After admission, the patient received conservative treatment using atorvastatin plus low-dose dexamethasone. After 2 weeks of inpatient therapy, head MRI revealed that the subdural mass had grown, with significant enhancement of the dura mater (**Figure 4B**). A substantial leukocyte abnormality was detected during the treatment period, and a diagnosis of AML was made on the basis of bone marrow aspirate examination (**Figure 1C**). Conservative treatment using atorvastatin plus dexamethasone was switched to periodic chemotherapy using cytarabine plus decitabine for over 4 months. Surprisingly, the subdural hematoma was significantly absorbed after completion of chemotherapy (**Figures 4C, D**) and the patient’s symptoms disappeared with complete remission of the AML (**Figure 1D**). This course suggested that the patient had CSDH that was related to leukemia encephalopathy. The 5-month follow-up MRI findings (**Figure 4E**) revealed no subdural hematoma and the patient did not experience any severe side effects during the treatment period.

## Discussion

The development of CSDH is related to chronic inflammation in the subdural space that induces the formation of highly permeable capillaries, which leads to continuous blood leakage and accumulation in the subdural space (4–6). Among the cases we reported, the CSDH in Patients 1 and 2 might be related to incidental cerebral trauma during leukemia treatment, which could have been accompanied by the inflammation-related formation of immature blood vessels, leading to CSDH. In Patient 3, dural metastasis may have been responsible for the subdural mass, as dural invasion of leukemia can occasionally cause malignant subdural hematoma (8). Our laboratory findings revealed low platelet counts in all 3 patients (**Supplement Table 1**), which is concordant with the relationship between abnormal coagulation function and the development of CSDH (18–20). In Patients 1 and 2, atorvastatin plus low-dose dexamethasone treatment promoted absorption of the CSDH and alleviated the patients’ neurological symptoms with no adverse events occurred and no recurrence during follow-up. Thus, this combination may be a safe and effective treatment for patients with leukemia and conventional CSDH.

Atorvastatin treatment for CSDH is based on the concept that statin treatment is effective for subdural masses (13). The anti-inflammatory effects of statin may play an important role in promoting the absorption of CSDH. For example, our previous studies revealed that atorvastatin treatment of rats with subdural hematoma led to decreased expression of IL-6, IL-8, and TNF-α, which contribute to the development of CSDH, and increased the number of anti-inflammatory regulatory T-cells (15–17). While atorvastatin alone initially improved Patient 1’s neurological symptoms, there was no clear CSDH absorption, which was subsequently observed after the addition of dexamethasone. This result agrees with the finding from a phase II randomized proof-of-concept trial, which indicated that atorvastatin plus low-dose dexamethasone was more effective than atorvastatin monotherapy for accelerating CSDH absorption. That study indicated that atorvastatin enhanced the levels of circulating endothelial progenitor cells, which are essential for the formation and maturation of blood vessels, and promoted the maturation of subdural leakage capillaries, whereas dexamethasone strengthened these effects (14). *In vitro* research showed that subdural hematoma could destroy endothelial cell junction by reducing the expression of transcription factor KLF2, while statin could promote the expression of KLF2 to improve endothelial connection. And the effect of statins on promoting the expression of KLF2 was enhanced when combined with low-dose dexamethasone (21).

Patient 3 had an interesting course, as the subdural hematoma was not effectively absorbed during the early stage of treatment using atorvastatin plus dexamethasone. Furthermore, a new subdural hemorrhage during this period led to an increase in the CSDH. However, the patient was immediately switched to chemotherapy after the diagnosis of AML, which resulted in resolution of the leukemia after 6 weeks and rapid absorption of the subdural mass at the same time based on the MRI findings. Thus, the patient likely had leukemia encephalopathy rather than conventional trauma-related CSDH. Furthermore, enhancement of the dura mater was observed, which confirmed that there was metastasis of the leukemia cells. Although there was no history of trauma in the 3^rd^ patient, dural invasion by leukemic cells may have been the source of subdural masses (8). A recent rat-based study has indicated that the dural lymphatic vessels are an important channel for subdural hematoma drainage to the extracranial space (22). Whereas tumor metastasis can lead to dysfunction of lymphatic drainage (23), leukemia cells invading the patient’s dura mater may occlude the meningeal lymphatic drainage pathway, which can reduce the absorption of CSDH. Effective chemotherapy may restore this drainage pathway, with relief from leukemia. Therefore, Patient 3 may not have initially responded to atorvastatin treatment because meningeal metastasis may have caused dural lymphatic drainage dysfunction, which suggests that atorvastatin-based conservative treatment alone may not be effective for patients with subdural masses related to leukemia encephalopathy. Remission of leukemia by chemotherapy allows hematomas associated with leukemia encephalopathy to be absorbed.

After treatment, subdural hematoma of 3 patients were significantly absorbed, and no recurrence was found in the follow-up. Patients 1 and 2 were satisfied with our statins based treatment, and patient 3 highly appreciated our correct diagnosis and chemotherapy regimen.

## Conclusion

We encountered 3 men with myeloid leukemia and CSDH, who experienced different responses to conservative treatment using atorvastatin plus low-dose dexamethasone. Two of the patients had conventional trauma-related CSDH, and the conservative treatment promoted subdural hematoma absorption and improved their neurological symptoms with no adverse effects. However, the subdural hematoma in the patient who was diagnosed as having leukemia encephalopathy did not respond to statin-based treatment. After chemotherapy, leukemia resolved and the subdural mass disappeared completely. Thus, atorvastatin plus low-dose dexamethasone may be an effective conservative treatment for conventional trauma-related CSDH in leukemia patients but not for subdural masses caused by leukemia encephalopathy.

## Data Availability Statement

The original contributions presented in the study are included in the article/**Supplementary Material**. Further inquiries can be directed to the corresponding authors.

## Ethics Statement

Written informed consent was obtained from the individual(s) for the publication of any potentially identifiable images or data included in this article.

## Author Contributions

JY analyzed dates and wrote article. YL, XL, MN, WJ, YF, HW, and TX collected patients information. WQ, CG, JH, and SA offered writing advices. YR and QZ helped to design figures. JZ and RJ revised papers and provided financial support. All authors contributed to the article and approved the submitted version.

## Funding

This study was supported by the National Natural Science Foundation of China (No.81671221), the Clinical Study of Tianjin Medical University (No. 2017kylc007) and the Science and Technology Project of Tianjin (No.19YFZCSY00650).

## Conflict of Interest

The authors declare that the research was conducted in the absence of any commercial or financial relationships that could be construed as a potential conflict of interest.
