# Supplementary figures and images for: Atorvastatin Plus Low-Dose Dexamethasone May Be Effective for Leukemia-Related Chronic Subdural Hematoma but Not for Leukemia Encephalopathy: A Report of Three Cases
Source: Front Oncol. 2021 Jul 15;11:628927. doi: 10.3389/fonc.2021.628927 (PMC8320332; doi:10.3389/fonc.2021.628927)

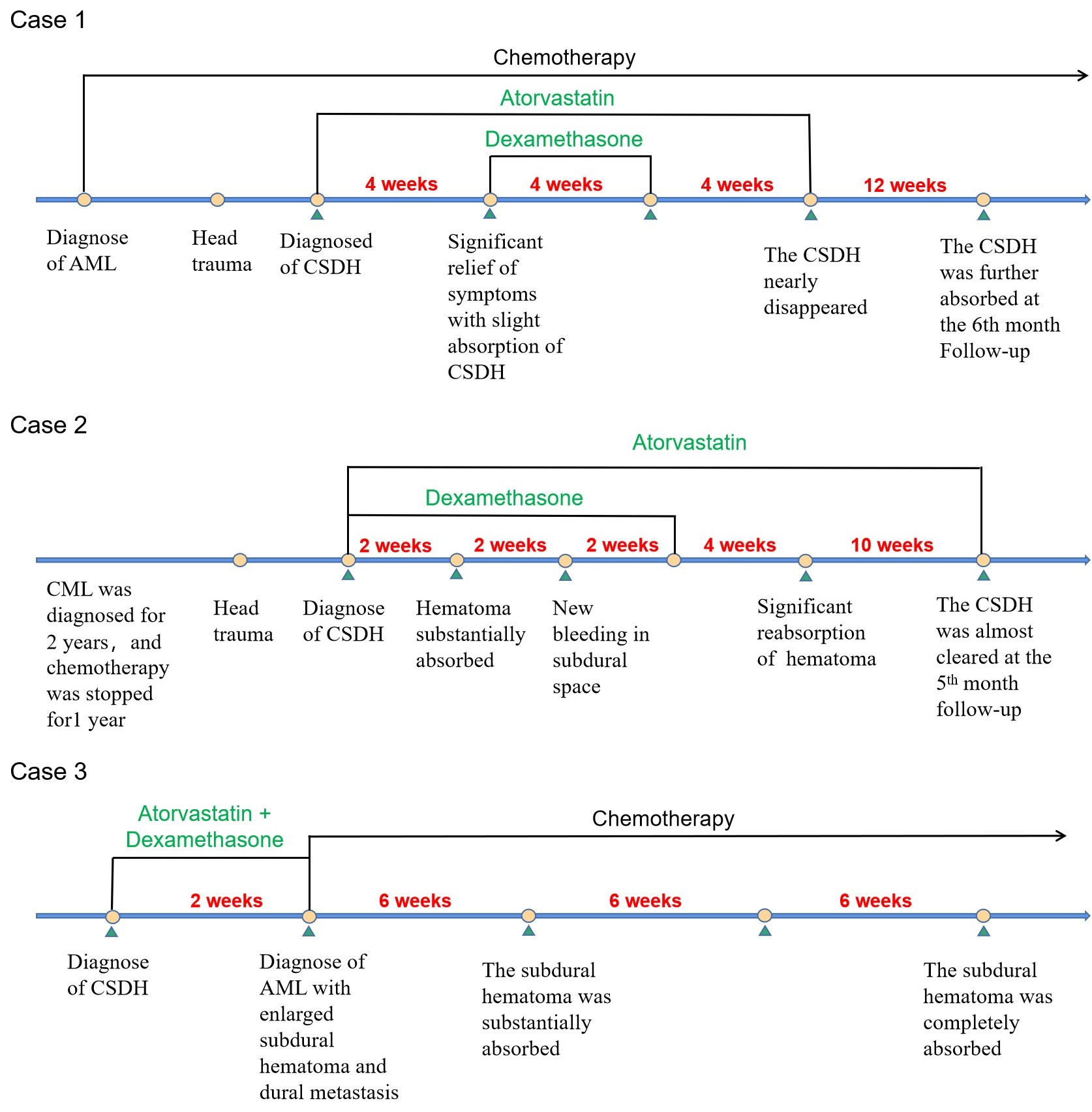

Supplement: Supplementary file 1 [file Image_1.jpeg]
