# Supplementary material for: Atorvastatin Plus Low-Dose Dexamethasone May Be Effective for Leukemia-Related Chronic Subdural Hematoma but Not for Leukemia Encephalopathy: A Report of Three Cases
Source: Front Oncol. 2021 Jul 15;11:628927. doi: 10.3389/fonc.2021.628927 (PMC8320332; doi:10.3389/fonc.2021.628927)
Supplement: Supplementary file 2 [file Table_1.doc]

**Supplement Table 1.** Clinical characteristics of and conservative treatment processes in the three patients.

| **Case**  **No./Sex** | **Age**  **yrs** | **Trauma History**  **(Y/N)** | | **Symptoms of CSDH** | **Leukemia Phenotype** | **Dura mater Metastasis**  **（Y/N）** | **Platelet counts of CSDH diagnosed** | **Chemotherapy**  **(Y/N)** | **Treatment with atorvastatin** | **Treatment with**  **dexamethasone** | **Therapeutic outcome of CSDH** | **Recurrence at follow-up**  **(Y/N)** |
| --- | --- | --- | --- | --- | --- | --- | --- | --- | --- | --- | --- | --- |
| 1/M | 28 | Y | Headache; confusion | | AML | N | 42*10^9^/L | Y | 20 mg/day for 12 weeks | 4 weeks **#** | Symptoms disappeared completely；CSDH almost absorbed | **N** |
| 2/M | 72 | Y | Headache; dizziness; weakness of left limb | | CML | **_** | 62*10^9^/L | N | 20 mg/day for 20 weeks | 6 weeks **#** and 2.25 mg/day for an additional 2 weeks | Symptoms disappeared completely；CSDH almost absorbed | N |
| 3/M | 68 | N | Headache; dizziness | | AML | Y | 45*10^9^/L | Y | 20 mg/day for 2 weeks | 2.25 mg/day for 2 weeks | Symptoms and CSDH disappeared completely | N |

CSDH：Chronic Subdural Hematoma； AML: Acute Myeloid Leukemia；CML: Chronic Myeloid Leukemia

# Daily 2.25 mg doses of dexamethasone orally for the first 2 weeks, followed by daily doses of 1. 5 mg of dexamethasone for 1 week and then daily doses of 0.75 mg of dexamethasone for 1 week.

Normal range of platelet counts: 100-300 × 10^9^/L.
